# Correlative multimodal imaging for microscale spatial mapping of collagen-gene activity interactions in human tissues

**DOI:** 10.1038/s44303-026-00149-8

**Published:** 2026-03-23

**Authors:** Riccardo Scodellaro, Martina Mietto, Alessandra Ferlini, Frauke Alves

**Affiliations:** 1 Translational Molecular Imaging, Max Planck Institute for Multidisciplinary Sciences, Göttingen, Germany; 2 Unit of Medical Genetics, Department of Medical Sciences, University of Ferrara, Ferrara, Italy; 3 Department of Haematology and Medical Oncology, University Medical Center Göttingen, Göttingen, Germany; 4 Institute for Diagnostic and Interventional Radiology, University Medical Center Göttingen, Göttingen, Germany

**Keywords:** Biological techniques, Computational biology and bioinformatics, Diseases

## Abstract

Understanding how gene activity relates to other biological structures is critical to investigate tissue remodeling processes, disease, and regeneration. RNAscope in situ hybridization assay provides single-molecule detection of targeted transcripts, while label-free multiphoton microscopy enables high-resolution, quantitative imaging of extracellular matrix collagen. These modalities have not previously been combined to extract spatially resolved correlations between molecular and structural features within the same tissue section. Here, we introduce correlative multimodal imaging that integrates RNAscope with Second Harmonic Generation microscopy to align transcript localization with quantitative metrics of collagen architecture at microscale resolution. We applied this approach to human skeletal muscle biopsies of healthy and diseased patients, affected by Duchenne Muscular Dystrophy. Applying our workflow, we observed that, in this proof-of-concept, regions enriched in specific dystrophin transcripts (targeting exons 37–42 and 63–75) are associated with localized increases in collagen fiber length and density, suggesting a potential spatial correlation between dystrophin transcript distribution and collagen organization. This workflow enables microscale integration of molecular and structural data. Moreover, it can be readily extended to diverse tissues, targets, and disease contexts, providing a versatile platform for a deeper spatial biomarker discovery, fibrosis and regeneration studies, microscale evaluation of morphological effects on tissue of transcript-based therapies.

## Introduction

The spatial relationship between gene activity and tissue remodeling is a key driver of tissue development, disease progression, and therapeutic response. Processes such as fibrosis, cancer invasion, and neuromuscular degeneration involve coordinated changes in transcript dynamics and tissue architecture across micro- and macro-scales, yet these molecular and structural dimensions are rarely investigated together within the same tissue section.

RNAscope in situ hybridization (ISH) assay has emerged as a powerful technique to detect specific transcripts at single-molecule resolution. Based on a signal amplification system, RNAscope employs double Z-target probe pairs that hybridize specifically to target RNA sequences while minimizing background noise, enabling simultaneous visualization of transcript abundance, localization, and integrity within intact cells and tissues^1–3^. This approach has revealed that spatial localization of transcripts, whether in the cytoplasm, at specific organelles, or close to their transcriptional loci, can influence post-transcriptional regulation via mechanisms such as RNA binding protein interaction, localized translation, or RNA stability. These mechanisms suggest that transcript compartmentalization can have important implications for designing RNA-targeted therapies.^4,5^.

In parallel, imaging techniques have been extensively used to characterize collagen and tissue remodeling. Among these, multiphoton microscopy (MPM) has gained particular traction due to its label-free imaging capabilities and minimal sample preparation. By leveraging intrinsic nonlinear optical signals such as Second Harmonic Generation (SHG) from collagen and myosin, and Third Harmonic Generation (THG) from lipids, MPM provides high-resolution, quantitative information on ECM composition and organization without staining^6–11^. Because collagen, fat, and myosin are key structural components in many pathological processes, MPM has been successfully applied to diverse contexts ranging from cancer to corneal edema and neuromuscular disease^12–14^, and even coupled with new artificial intelligence methods^15^ to evaluate human biopsies^16^.

Due to its non-destructive nature, characterized by the use of unstained tissue and minimal manipulation, MPM is ideally suited for multimodal strategies. Its compatibility with approaches such as MALDI imaging^17^ and coherent anti-Stokes Raman scattering (CARS) microscopy^18^ has already demonstrated the potential to extract complementary molecular and structural features from the same specimen. However, while correlative SHG-based imaging strategies have been successfully combined with protein-level or biochemical modalities (e.g., immunofluorescence, MALDI, or vibrational microscopy), the direct integration of single-molecule RNA in situ hybridization with MPM on the same tissue section has not yet been explored at microscopic resolution.

With this study, we aim to enrich the correlative SHG imaging state-of-the-art by introducing a complementary multimodal workflow that couples RNAscope ISH assay with label-free MPM. As a proof-of-concept, we demonstrate this approach on human skeletal muscle biopsies, specifically samples affected by Duchenne muscular dystrophy (DMD), thereby showing its feasibility of aligning RNA transcripts signals with quantitative collagen metrics in clinically relevant tissue. DMD is not curable, and most patients face fatal complications by their third or fourth decade of life^19^. In this application, we selected DMD pathology since it is characterized by transcript dysregulation, resulting in the absence of functional dystrophin protein^20^. The loss of dystrophin compromises the integrity of the dystrophin-glycoprotein complex, leading to sarcolemmal instability, disrupted calcium homeostasis, altered signal transduction, and repeated cycles of muscle fiber degeneration and regeneration. These pathological events culminate in chronic inflammation and progressive replacement of muscle tissue with fibrotic and adipose deposits^21^, phenotypically expressed by progressive muscle weakness, loss of ambulation, cardiomyopathy, respiratory decline, and neurocognitive deficits due to the absence of brain-expressed dystrophin isoforms^22^. In this context, DMD gene transcription is a long and tightly regulated process, involving complex splicing mechanisms such as recursive and alternative splicing^23^. This results in a wide set of isoforms, including three full-length variants (Dp427b, Dp427m, Dp427p) and several shorter, tissue-specific isoforms such as Dp260, Dp140, Dp116, Dp71, and Dp40^24,25^. However, the state-of-the-art understanding of dystrophin transcript dynamics, especially in terms of their spatial distribution and subcellular localization, remains limited.

By integrating both macroscale (whole-sample) and microscale (tens of µm) analyses, this flexible method enables the identification of spatially resolved correlations between transcript localization and collagen structure, providing a broadly applicable platform for biomarker discovery and studies of tissue remodeling.

## Results

Here, we show both the analysis that can be performed by leveraging the two methods independently, as well as a spatially-enhanced analysis of the skeletal muscles, which is a technical validation of our framework, correlating images obtained by the two techniques and applying the proposed proximity analysis. Finally, we provide an analysis of potential biases.

### Standalone RNAscope ISH data analysis

**Fig. 1:**
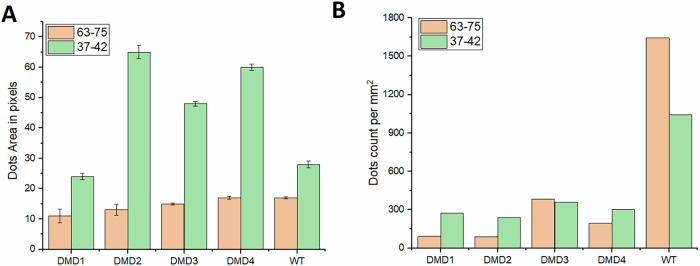
Global quantification of RNAscope-derived features across DMD and WT samples. Panel **A** shows the mean area (in pixels) of detected dots for probe targeting exons 37–42 (green) and 63–75 (orange), while **B** reports the number of detected dots per mm² for each sample and probe. Error bars indicate standard error of the mean (SEM).

To assess transcript expression independently of tissue morphology, we first analyzed the brightfield images obtained from RNAscope ISH assay. The transcripts appear as distinct red dots, which can be quantified based on their number and size. RNAscope assay provides localized molecular information, enabling the direct evaluation of transcript presence and spatial distribution. Figure 1 summarizes two key quantitative metrics derived from the RNAscope images: the mean area of each detected transcript-associated dot (in pixels), and the number of dots per mm², normalized by the sample area, which reflects the density of the dots within the tissue.

Across the analyzed DMD and WT samples, the probe targeting exons 37–42, primarily detects the Dp427m isoform in muscle, was associated with larger dots than the probe targeting exons 63–75, which detects both Dp427m and Dp71 (Fig. 1A). Differences in dot area reflect relative variations in RNAscope signal extent and local transcript accumulation. Although Dp71 is typically absent in mature muscle fibers, it may still be present at low levels as a secondary consequence of muscle degeneration and regeneration. In each independent sample, the mean area of 63–75-associated dots was below 20 pixels, whereas dots associated with 37–42 exceeded 24 pixels. These results confirm previous findings^3^, suggesting that the observed differences in signal intensity may arise from different conformations of the full-length Dp427m transcript, which may be partially spliced or enriched in ribonucleoprotein complexes.

In terms of dot count per mm^2^ (Fig. 1B), the WT sample displays higher transcript density, with over 1600 dots/mm² for the 63–75 probe and approximately 1100 dots/mm² for the 37–42 probe. In contrast, all DMD samples show reduced transcript counts, regardless of the probe used. For instance, DMD1 and DMD2 have fewer than 300 dots/mm² for either probe. This observation is consistent with the expected downregulation of dystrophin mRNA expression reported in dystrophic skeletal muscle.

### Standalone multiphoton microscopy data analysis

**Fig. 2:**
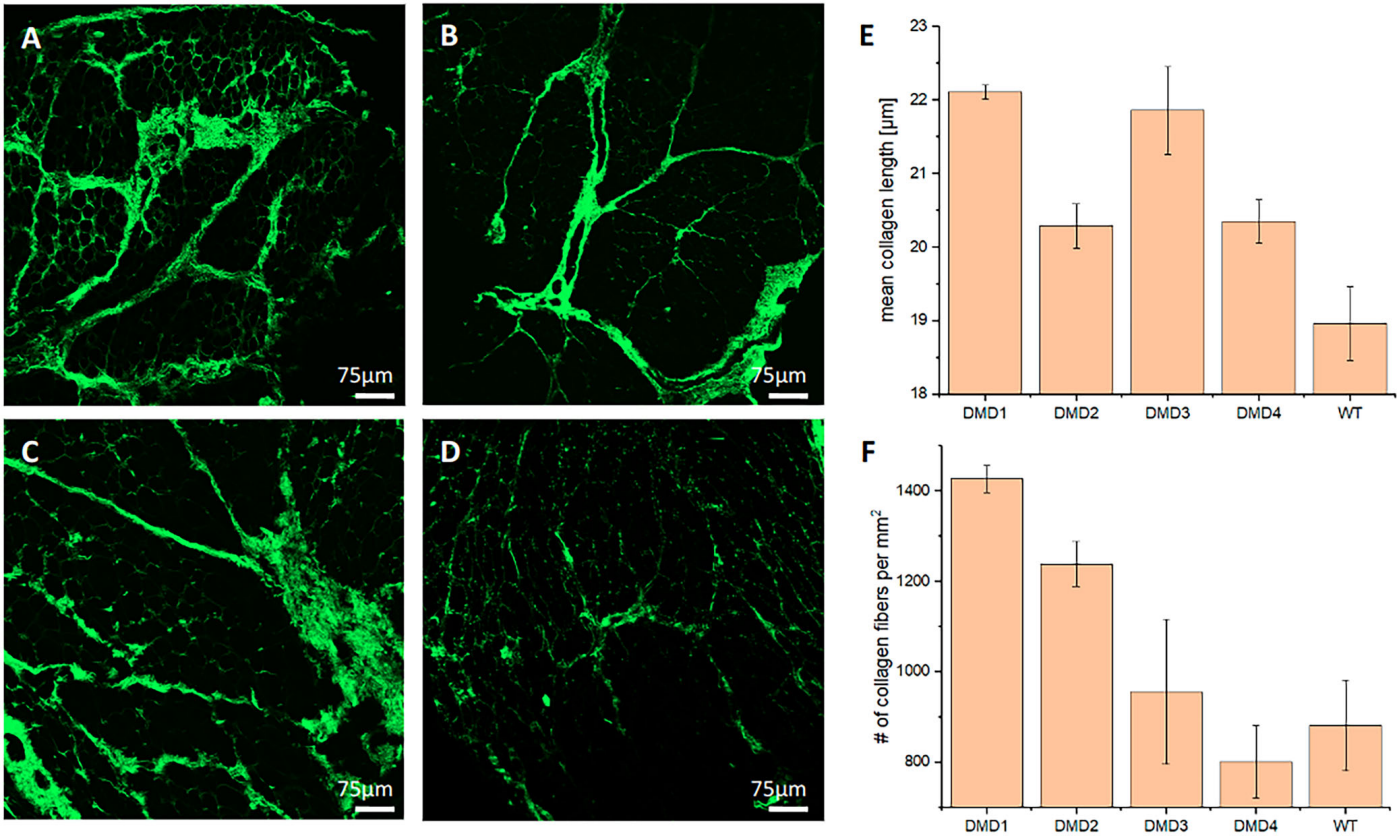
Collagen architecture assessment using SHG-based multiphoton microscopy. Panels **A**–**C** show representative regions of interest (ROIs) from three different DMD-affected muscle sections, while **D** reports an ROI from the WT muscle section. **E** Demonstrates the mean collagen fiber length in each sample, while **F** shows the number of collagen fibers per mm², normalized to tissue area. In both bar graphs, data represent the average of two slides per sample (targeting exons 37–42 and 63–75); error bars indicate standard error of the mean (SEM).

To evaluate tissue architecture independently of transcript expression, we performed the analysis of label-free imaging of collagen in skeletal muscle sections using multiphoton microscopy. By exploiting the SHG signal, we visualized specifically collagen fibers and quantify their features and organization across all samples. The results of this collagen-limited image analysis are reported in Fig. 2.

The SHG images from DMD samples (Fig. 2A–C) reveal widespread and heterogeneous collagen deposition throughout the tissue, with dense bundles crossing multiple regions. In contrast, the WT sample (Fig. 2D) exhibits minimal intramuscular collagen presence, with more uniform distribution. This visual distinction reflects the typical fibrotic remodeling observed in DMD pathology. To quantify these differences, we computed two global metrics across each sample: the mean fiber length and the mean fiber density (i.e., number of fibers per unit area). As shown in Fig. 2E, within this limited cohort, DMD samples displayed longer collagen fibers relative to the WT control. The WT control shows lower mean fiber length (19.0 ± 0.5 µm), while DMD1 reaches values above 22 µm. Fiber density, reported in Fig. 2F, exhibits high variability across DMD samples. DMD1 shows the highest number of collagen fibers (~1400 fibers/mm²), while DMD4 has values closer to those of WT (881 ± 88 fibers/mm²).

Since RNAscope does not alter collagen morphology or interfere with SHG acquisition, both slides, despite being treated with different probes (targeting exons 37–42 and 63–75), were considered comparable and analyzed together. The small standard errors confirm consistency between replicates and support the robustness of the collagen quantification pipeline.

### Registration process

**Fig. 3:**
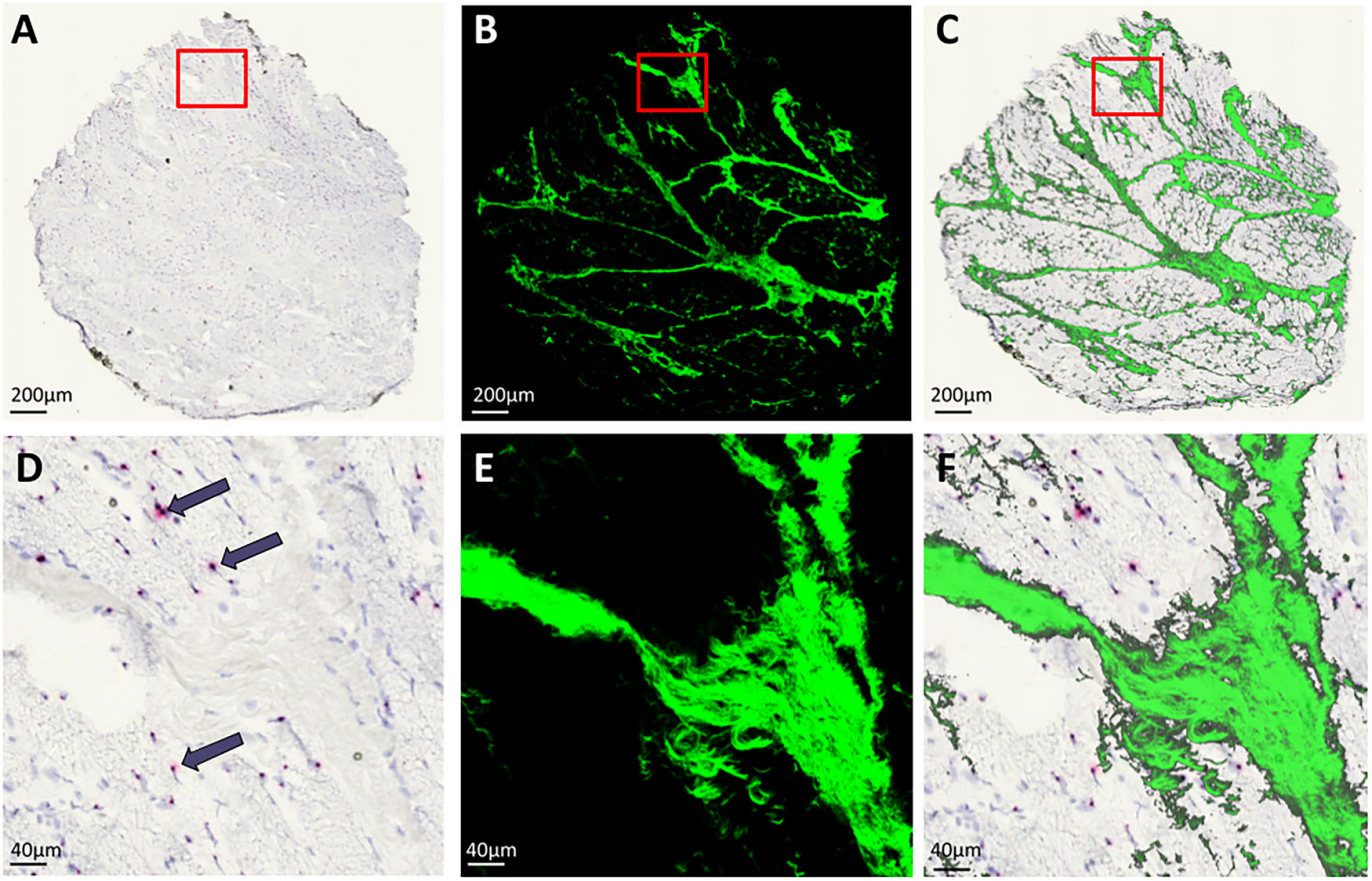
Registration of brightfield and multiphoton microscopy images from a DMD muscle section hybridized with RNAscope probe targeting exons 37–42. Panel **A** shows the brightfield scan of the sample, while **B** reports the corresponding SHG image acquired via multiphoton microscopy and **C** depicts the overlay of the two registered images. Panels **D**–**F** are zoomed-in views of the red-boxed areas present in (**A**–**C**), respectively. **D** Each single DMD transcript is represented as a distinct red dot (violet arrow). Sample stained with Gill’s Hematoxylin.

The registration process allowed pixelwise colocalization of DMD transcripts and collagen fibers. A representative example of this process is shown in Fig. 3. In the brightfield image (Fig. 3A), signals from the DMD transcript appear as distinct dots. As shown in the zoomed-in view (Fig. 3D), the signals are clearly identifiable; however, the collagen structure is barely visible due to the absence of specific staining. Indeed, stains such as Masson’s Trichrome, commonly used to visualize collagen, were not applied in this context.

Here, the SHG image (Fig. 3B) provides detailed visualization of the collagen architecture, as further highlighted in the magnified view (Fig. 3E). Moreover, these images show that RNAscope processing does not compromise SHG signal acquisition, a crucial aspect to demonstrate the feasibility of the proposed multimodal correlative approach. Importantly, since both imaging modalities were applied sequentially to the same mounted slide without any intermediate manipulation, tissue deformation was minimal for accurate and straightforward image registration. The obtained final image after data fusion (Fig. 3C) demonstrates a satisfactory spatial alignment between the two images. As shown in the overlay (Fig. 3F), regions of interest (ROI) are well co-localized, confirming that the RNAscope ISH assay does not interfere with SHG acquisition and that the tissue structure remains stable throughout the dual-imaging process.

### Macroscale spatial analysis by coupling RNAscope and multiphoton microscopy

Following image registration, the integration of RNAscope ISH assay with SHG-based multiphoton microscopy enables spatial correlation of transcript expression and collagen microarchitecture within the same tissue section. A major advantage of this approach lies in its ability to overlay molecular and structural features without introducing tissue artifacts, as both imaging modalities are applied sequentially on the same slide without intermediate handling or tissue manipulation.

**Fig. 4:**
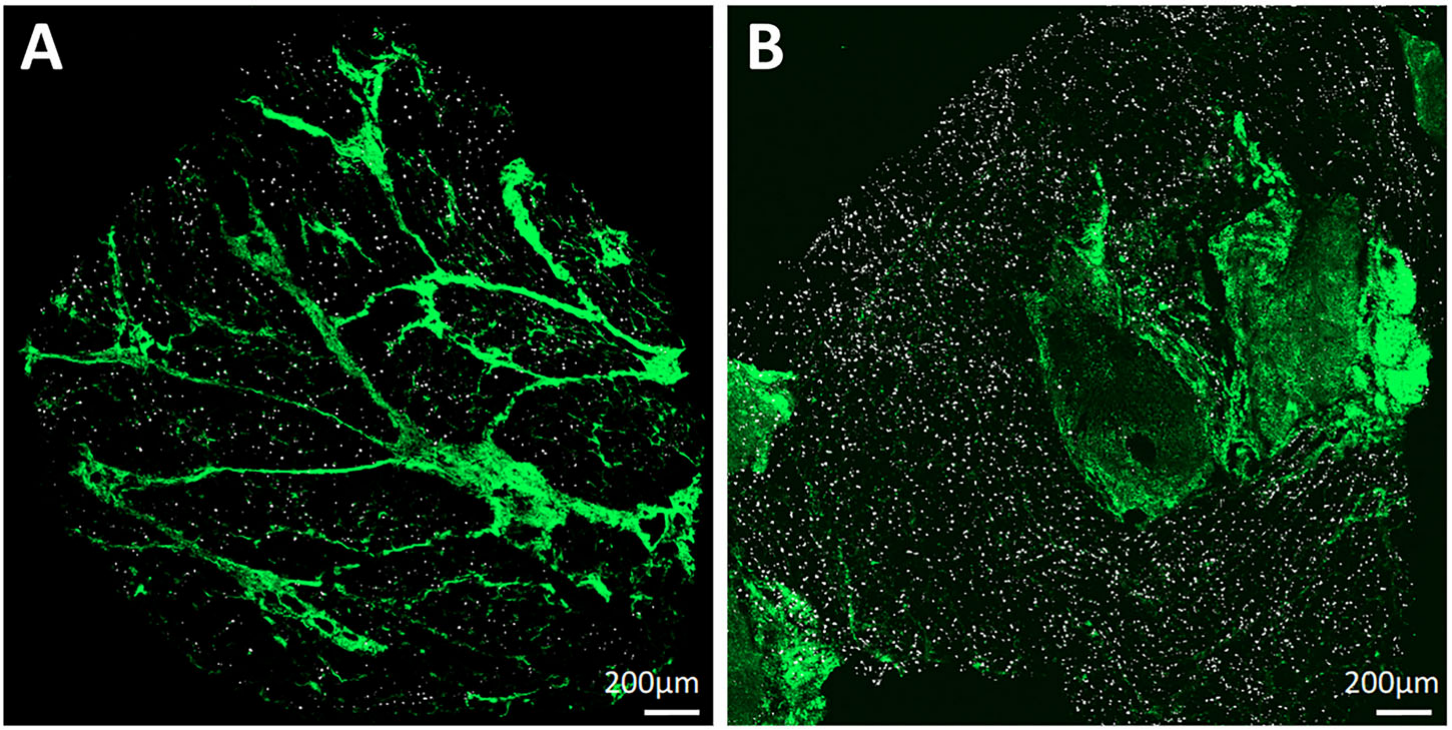
Human skeletal muscle sections of DMD and WT tissue samples after superposition of SHG signal (in green) and the binary masks reporting the dots extracted from RNAscope (in white). Panel **A** shows a DMD sample, with a more distributed and complex collagen architecture, while **B** reports the WT section, where a higher number of dots is visible.

In Fig. 4, we show the overlay of RNAscope signals (in white) and SHG signals from collagen (in green) for representative DMD and WT tissue sections. The DMD sample (Fig. 4A) shows a widespread, disorganized network of intramuscular collagen fibers, while the WT section (Fig. 4B) exhibits a very limited amount of intramuscular collagen and a more regionally compartmentalized collagen architecture. Consistent with earlier quantification (Fig. 2B), the WT section displays a visibly higher density of RNAscope-positive dots, confirming high transcript presence compared to the DMD samples we analyzed.

**Fig. 5:**
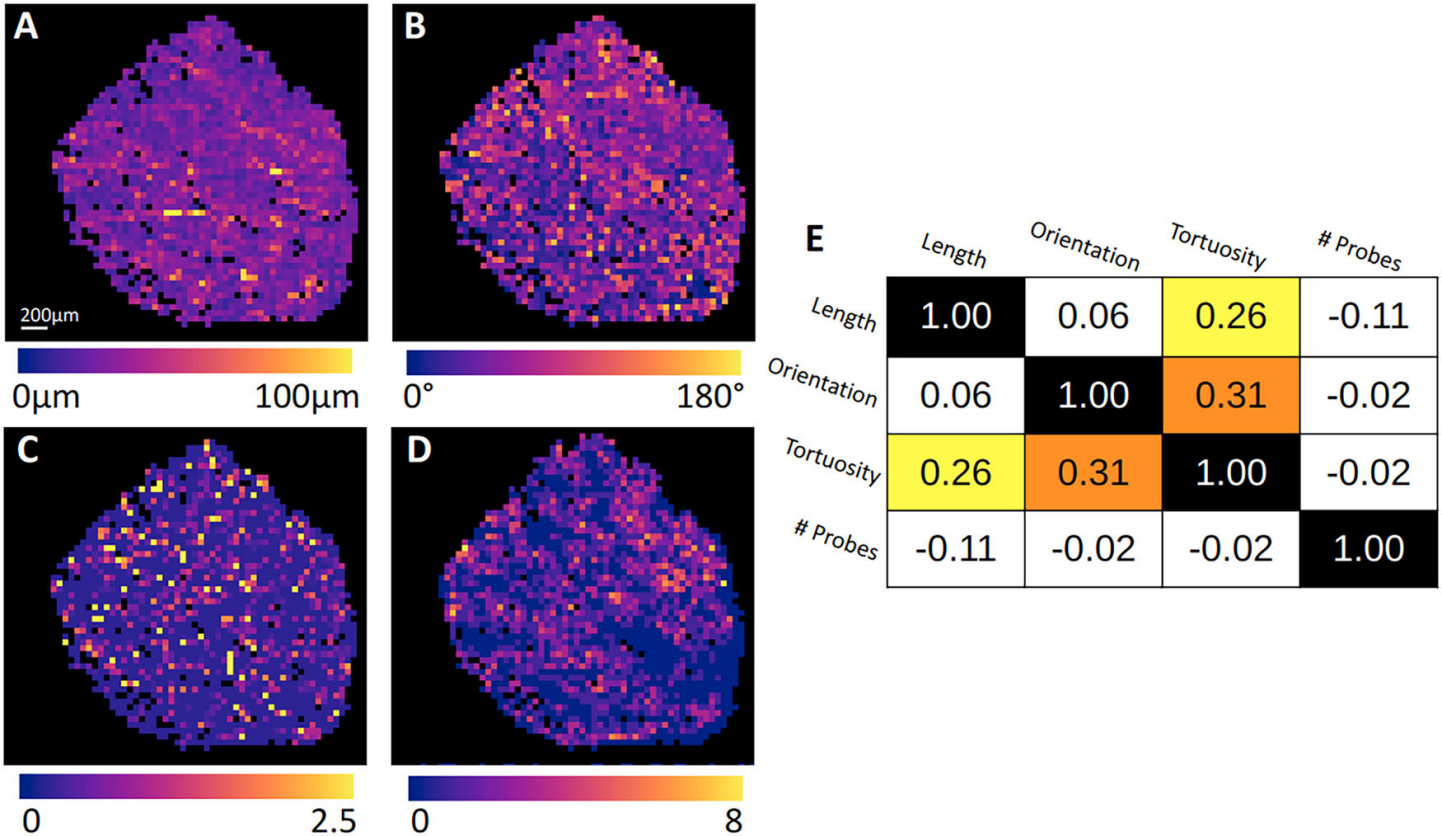
Grid-based macroscale analysis of collagen morphology and transcript signal distribution in DMD skeletal muscle. Show the grid maps representing three morphological features of collagen fibers computed from SHG images: **A** mean fiber length, **B** mean fiber orientation, and **C** mean fiber tortuosity. **D** reports the grid map of probe signal density derived from RNAscope analysis targeting exons 37–42, showing the number of detected transcript-associated dots per 100 × 100 pixel regions. Panel **E** shows the Spearman correlation matrix demonstrating the monotonic correlation between collagen features and probe density across the entire sample. Yellow and orange boxes indicate weak to moderate positive correlations (ρ < 0.4), while white indicates negligible correlations.

Beyond qualitative assessments, we performed a quantitative spatial analysis by subdividing the sample into a uniform grid of 100 × 100 pixel regions. For each grid unit, we computed average values of the three collagen-related morphological features we selected (fiber length, orientation, and tortuosity), derived from SHG images, as well as the density of RNAscope-positive dots. This allowed spatial colocalization of molecular and structural metrics across the entire tissue. Figure 5 presents the resulting spatial maps and correlation matrix.

Maps for collagen fiber length (Fig. 5A), orientation (Fig. 5B), and tortuosity (Fig. 5C) reveal substantial heterogeneity across the DMD sample. Notably, longer collagen fibers tend to form structured bundles bridging dense fibrotic regions, while shorter fibers dominate less populated areas. Tortuosity appears higher in the central part of the tissue section, suggesting localized architectural distortion.

The spatial distribution of transcript signal (Fig. 5D) also shows regional variability. The upper-right quadrant exhibits high probe density, while deep blue zones represent regions with minimal transcript signals. These patterns suggest non-uniform transcript activity across the tissue, potentially reflecting regional differences in pathological remodeling or probe accessibility.

To explore potential spatial correlations, we computed Spearman correlation coefficients between all extracted parameters on a region-wise basis (Fig. 5E). Collagen tortuosity shows low-to-moderate positive correlations with both fiber length (*ρ* = 0.26) and orientation (*ρ* = 0.31), suggesting that longer, more directionally aligned fibers may also exhibit increased path irregularity. However, probe density shows no substantial monotonic correlation with any collagen feature (|*ρ* | < 0.11), indicating that at this macroscopic scale, transcript abundance does not strongly co-vary with collagen structure.

These results suggest that although macroscopic maps reveal meaningful regional variation in both collagen morphology and transcript signal, their interrelation may be present for smaller resolutions. Therefore, higher-resolution, microscale analysis may be required to uncover more subtle spatial relationships.

### Microscale spatial analysis: technical validation of the proposed framework

**Fig. 6:**
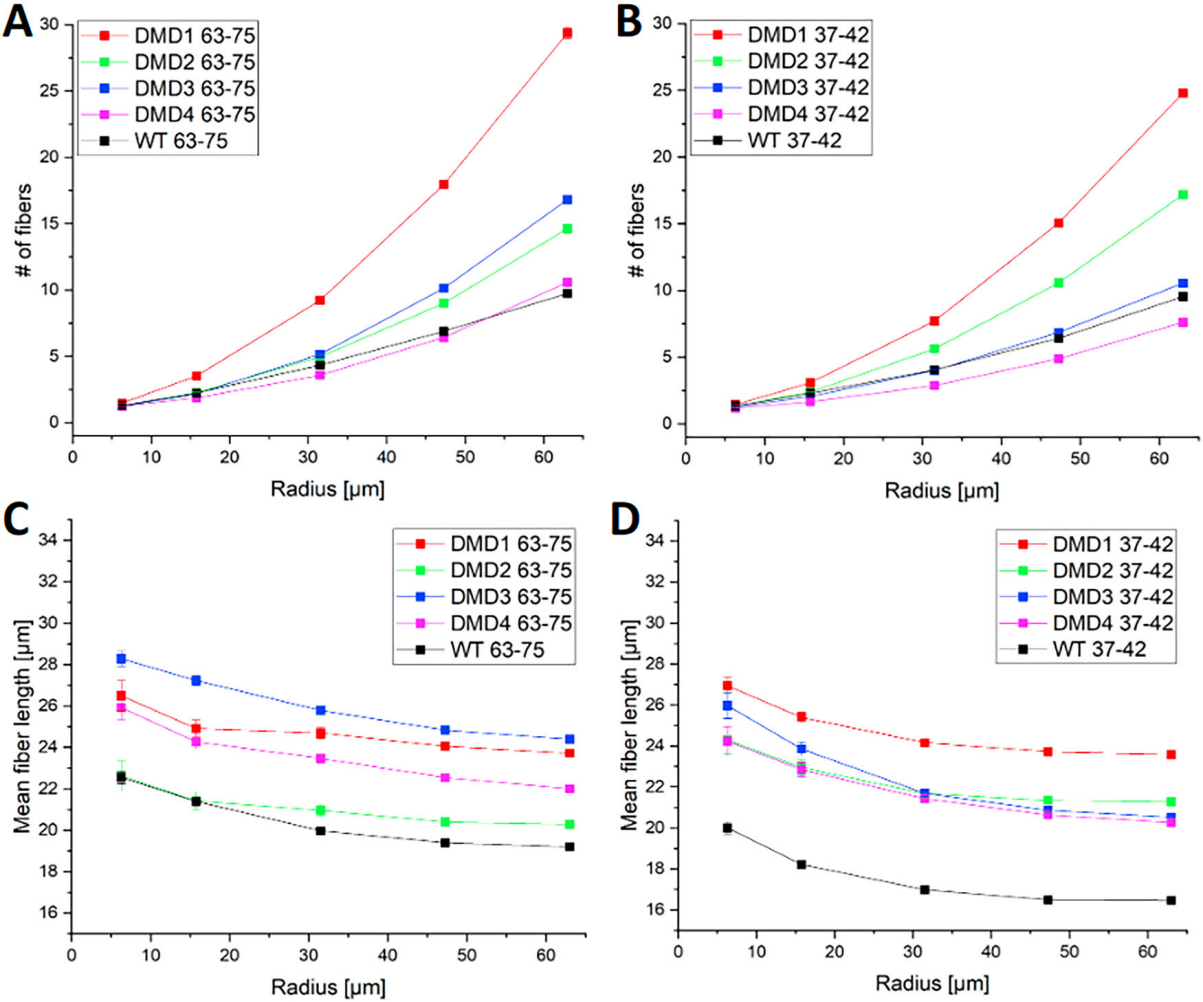
Microscale proximity analysis of collagen features around transcript signals. Show the mean number of collagen fibers detected within concentric circular regions centered on RNAscope-positive dots, as a function of radius, for probes targeting exons 63–75 (**A**) and 37–42 (**B**). Report the mean collagen fiber length as a function of distance from RNAscope-positive dots, for probes targeting exons 63–75 (**C**) and 37–42 (**D**). Each curve represents a single sample, with DMD and WT samples color-coded consistently across panels. Error bars represent the standard error of the mean (SEM). The analysis was performed for radii ranging from 6.3 to 63 µm.

**Fig. 7:**
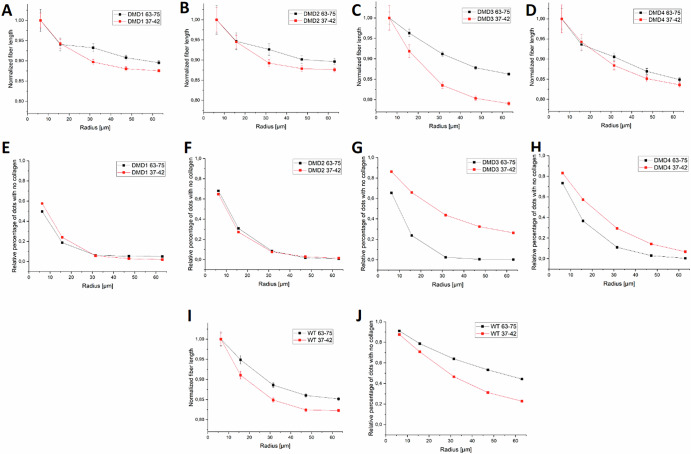
Normalized collagen fiber length profiles and collagen co-localization around transcript signals. Panels **A**–**D** show normalized mean collagen fiber length as a function of distance from transcript-associated dots in each DMD sample, for probe targeting exons 63–75 (black) and 37–42 (red). **I** Shows the same metric for the WT sample. Values are normalized to the fiber length at the smallest radius (6.3 µm) to highlight relative decay profiles. **E**–**H** Report the percentage of transcript-associated dots lacking any collagen fibers in their surroundings, across increasing radii, for each DMD sample. **J** Shows the same metric for the WT sample. A dot is considered “isolated” if no collagen fiber is detected within the defined radius. This analysis reveals differences in spatial proximity between transcripts and collagen fibers across disease states and probe targets. Error bars indicate standard error of the mean (SEM).

While the macroscale analysis revealed limited correlation between transcript abundance and collagen architecture, the proposed multimodal imaging strategy also enables a finer, microscale investigation. We implemented our novel proximity analysis described in the Methods section to assess local correlation between RNAscope-positive transcript signals and surrounding collagen features. Specifically, we (i) compared DMD and WT samples (Fig. 6), and (ii) investigated whether different transcript targets (exons 37–42 vs. 63–75) show distinct spatial collagen associations within the same sample (Fig. 7). For both analyses, we used concentric regions centered on each transcript dot, with radii ranging from 10 to 100 pixels (corresponding to approximately 6.3 to 63 µm).

Figure 6 presents the results of this proximity-based analysis. In Fig. 6A, B we show the mean number of collagen fibers found within increasing distances from the transcript-associated dots for each sample, using probes targeting exons 63–75 and 37–42. As expected, fiber count increases with distance, reflecting the expansion of the analyzed area. The rate of this increase is consistent between the two transcripts across all samples. Notably, DMD1 showed the highest fiber count even at short distances (e.g., >15 µm), consistent with the higher global collagen fiber density reported in Fig. 3F and suggesting an overall denser fibrotic matrix among the five analyzed samples.

Figure 6C, D depict the mean collagen fiber length as a function of distance from the probe signals. As previously observed (Fig. 3E), the WT sample is characterized by shorter collagen fibers compared to DMD tissues. However, interestingly, across all samples and both probes, a consistent trend emerges: collagen fibers tend to be longest in close proximity to RNAscope-positive signals and gradually decrease in length with increasing distance. This suggests a potential microscale phenomenon that can be captured by the proposed correlative approach: collagen organization and development may be influenced by local DMD transcript presence.

To further explore this observation, we normalized fiber length values in each sample to their respective maximum (always occurring at 6.3 µm), as shown in Fig. 7A–7D, I. This normalization allows a clearer comparison of the slope of fiber length decrease across distances. Across all samples, a gradual reduction is observed, with the most pronounced ones for the 37–42 probe, especially in DMD3. This steeper slope indicates greater local heterogeneity in collagen architecture around the Dp427m transcript, possibly reflecting differential roles or expression dynamics.

In Fig. 7E–H, J, we analyzed the percentage of transcript-associated dots lacking nearby collagen fibers as a function of increasing neighborhood radius. DMD1 and DMD2, characterized by higher global fiber density (Fig. 3F), show low percentages of isolated transcript signals: less than 10% of dots lack nearby collagen even at a 30 µm radius. Conversely, DMD3, DMD4, and WT samples show higher percentages of DMD transcript signals (i.e., dots) without adjacent fibers, particularly at small radii. Remarkably, in the WT sample, over 20% of transcript dots lack collagen fibers within a 60 µm radius, suggesting a more distant spatial correlation between transcript signals and collagen in this sample.

When comparing probes within individual samples, in DMD tissues, collagen fibers appear closer to transcript signals detected by the 63–75 probe compared to those associated with the 37–42 probe. In contrast, WT tissue showed the opposite trend, with collagen more closely associated with the 37–42 probe. This divergence may reflect differences in the localization of transcript regions detected by the probes. Although probe overlap prevents distinguishing isoforms in this particular study, the approach could reveal isoform-specific patterns if targets were non-overlapping. This spatial pattern can be explored in larger cohorts to assess its biological relevance.

Overall, this proximity-based microscale analysis shows that the proposed framework can quantify microscale spatial relationships between transcript localization and tissue architecture, intercepting potential different microenvironments within individual sections.

### Investigation of potential geometric and algorithmic biases

**Fig. 8:**
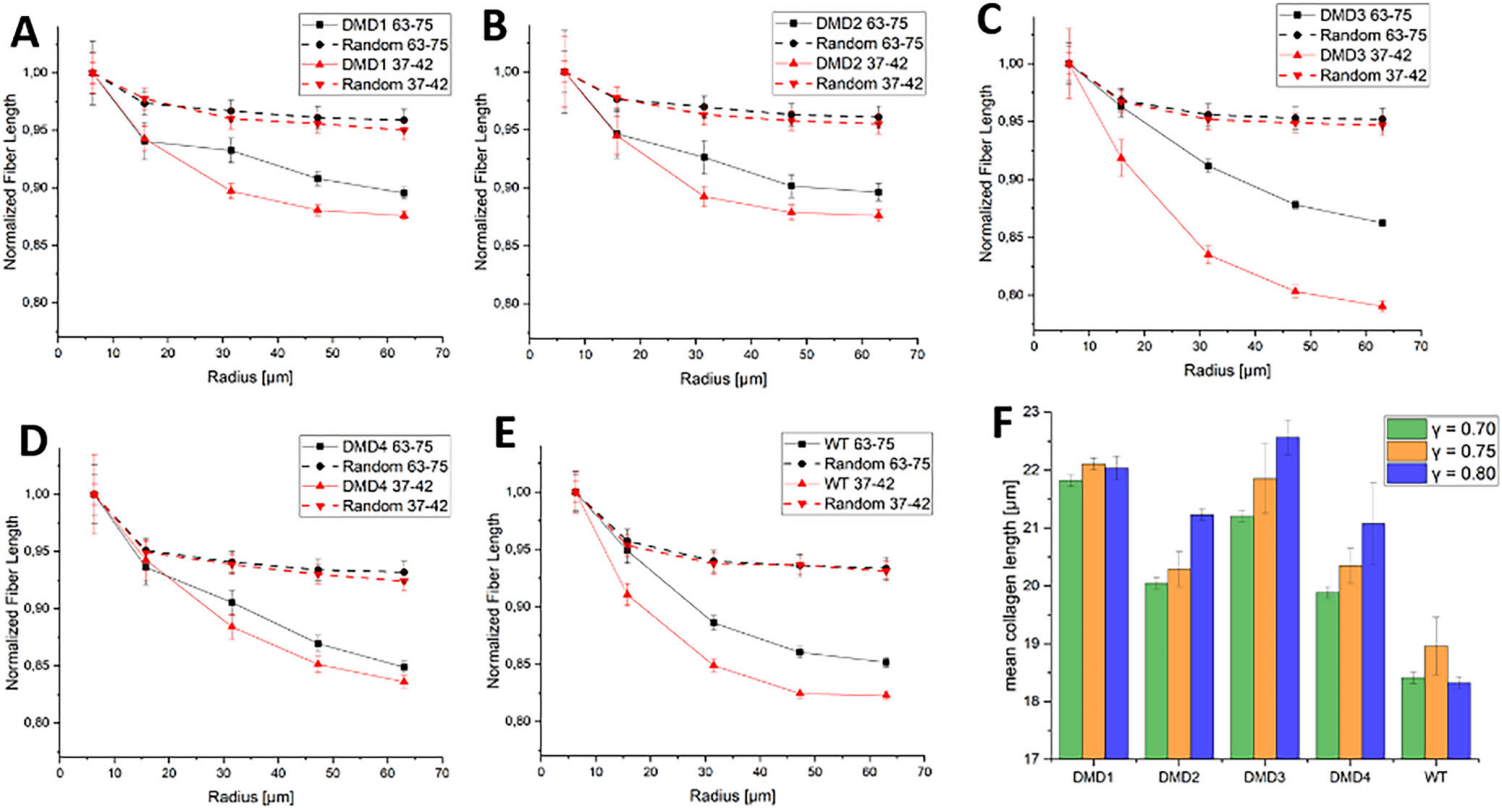
Assessment of geometric and algorithmic biases using randomized transcript dot distributions and controlled perturbations. **A**–**E** Report the normalized collagen fiber length as a function of distance from RNAscope-positive transcript dots for each sample. For each panel, curves obtained using the experimentally observed probes targeting exons 37–42 and 63–75 are compared with their corresponding randomized baselines. The experimentally observed curves display steeper slopes. **F** Means collagen fiber length for different γ corrections (left green box: γ = 0.70, center orange box: γ = 0.75, which correspond to the data of Fig. 3E, right blue box: γ = 0.80).

To assess whether the observed proximity-dependent trends between collagen fiber length and RNAscope-positive transcript dots could arise from geometric sampling bias, we implemented a null model control. For each tissue sample, 5000 transcript dot locations were randomized. Proximity analyses were then repeated using these randomized distributions to establish a baseline expectation under complete spatial randomness. In Fig. 8A–E, we compared the normalized fiber length obtained for each sample with the randomized baseline analysis. The slopes of the experimental proximity curves are greater than the ones obtained by considering random points. This indicates that, although geometric bias plays a moderate role to the proximity signal, the observed association between transcript dots and longer collagen fibers cannot be fully explained by random spatial placement and likely reflects a genuine connection.

While the randomized transcript dot analysis showed that geometric sampling bias alone cannot explain the observed proximity-dependent trends, we additionally assessed whether fiber segmentation procedure itself could introduce algorithmic bias. To this end, we assessed the stability of the leveraged network by performing a sensitivity analysis. In particular, we slightly modified image preprocessing parameters prior to the deep learning-based segmentation by considering two further γ corrections (0.70 and 0.80). These parameters slightly alter the SHG signal, which is the information leveraged by the network to segment collagen structures. We report the results on this test in Fig. 8F, showing that, in presence of these perturbations, slight discrepancies concerning the absolute fiber length value are present, in the order of ~1 µm. However, the general trends obtained by comparing the estimations on the different samples are preserved.

## Discussion

In this study, we present a correlative multimodal imaging framework that integrates RNAscope-based in situ hybridization with label-free multiphoton SHG microscopy to investigate potential spatial correlations between transcript localization and collagen morphology. By achieving pixelwise alignment of molecular and structural features in the same intact tissue section, this platform establishes a new methodological approach for high-resolution correlative analysis in spatial biology.

A strength of this approach lies in its ability to interrogate molecular–structural correlations at different spatial scales. At the macroscale, transcript abundance and collagen morphology appeared largely independent, reflecting the heterogeneity and complexity of the muscle tissue, as established in clinics. However, at the microscale, our proximity analysis suggests local associations, with longer collagen fibers in close proximity to RNAscope-positive transcript signals, both in control and diseased tissues. This correlation would remain undetected without the proposed multimodal integration. This emphasizes a central message: large-scale averages can hide biologically meaningful microscale dependencies, which are only revealed through correlative imaging at cellular and subcellular resolution.

The proof-of-concept application to DMD illustrates the future biological relevance of this approach, as fibrosis and transcript dysregulation are defining features of the disease. Importantly, isoform-specific differences emerged when comparing probes targeting distinct regions of the DMD gene, suggesting that different transcript variants may be associated with distinct microarchitectural patterns of collagen. The capability to spatially resolve isoform-specific remodeling signatures highlights the potential of the method to dissect transcript diversity in both physiological and pathological contexts. Despite the limited cohort, our proof-of-concept relies on human patient biopsies, rather than animal models or cell systems. This not only underscores the translational potential of the pipeline but also demonstrates its robustness in clinically relevant material, where sample availability and integrity often limit multimodal imaging strategies.

Although applied here to skeletal muscle, the broader implications of this pipeline extend across biomedical research. Fibrosis and ECM remodeling are fundamental to cancer progression, chronic inflammation, cardiovascular pathology, and organ regeneration^7,26^. The ability to map local transcript-related relationships with the morphological properties of other structures offers new opportunities for biomarker discovery and patient stratification, suggesting new potential subtypes characterized by different tissue architectures or treatment responses. Moreover, the method is inherently compatible with advanced modalities such as light-sheet^27^ or cleared-tissue multiphoton imaging for three-dimensional analysis^26^, and with label-free multiparametric approaches such as SLAM (Simultaneous Label-free Autofluorescence Multiharmonic) microscopy approach, which combines autofluorescence, SHG, and THG signals^28^. Integrating these modalities would enable a richer characterization of cellular and extracellular components in relation to gene expression. Coupling with artificial intelligence for automated feature extraction further expands the translational potential, enabling high-throughput analysis of clinical biopsies.

This study has limitations: it was based on a small cohort, reflecting the proof-of-concept nature of the work. The transcript-associated spatial observations were not treated as independent replicates, but averaged at sample level, preserving biological independence. Larger cohorts will be required in future studies to depict clear biological conclusions. The aim was not to establish definitive clinical insights, but rather to demonstrate the feasibility and power of the correlative pipeline. Future studies with larger patient cohorts, longitudinal sampling, and inclusion of multiple disease models will be essential to validate and generalize the observed spatial patterns. In addition, the possibility of expanding the approach beyond collagen to other ECM components, lipids, or intracellular proteins may provide a more comprehensive picture of tissue remodeling dynamics. An additional limitation is that nuclear versus cytoplasmic localization of RNAscope-positive transcripts was not explicitly resolved. As a result, the observed spatial associations cannot be attributed to specific subcellular transcript states, such as active transcription versus mature mRNA localization. Future extensions of this framework could incorporate nuclear segmentation (e.g., using immunofluorescent nuclear markers) to stratify transcript signals by subcellular compartment and investigate whether nuclear or cytoplasmic transcripts exhibit distinct spatial relationships with collagen morphological features. Although the RNAscope probes were designed to preferentially target regions associated with distinct isoforms, partial sequence overlap limits isoform-exclusive detection. Nevertheless, refining probe design or combining with other technologies could allow direct visualization of isoform-specific distributions, offering a powerful avenue to explore the spatial biology of dystrophin transcripts. Similarly, future extensions of this framework incorporating cell-type-resolved strategies would strengthen biological interpretation. For example, the use of immunofluorescent markers for fibroblasts and myogenic cells, or integrating multiplexed spatial transcriptomics approaches, would enable attribution of transcript signals to specific cell types. This would allow discrimination between transcripts expressed by ECM-producing fibroblasts and those arising from neighboring muscle fibers or inflammatory cells, thereby clarifying whether the observed spatial associations reflect direct cellular contributions or paracrine microenvironmental effects. From the algorithmic perspective, despite demonstrating sufficient stability of the collagen fibers segmentation, the reliability of the used neural network could be improved by performing fine tuning on manually annotated SHG signals, considering both regions characterized by sparser and denser collagen fibers.

By combining RNAscope ISH assays with multiphoton microscopy, we established a powerful and flexible platform that bridges transcript activity detection and structural imaging. This correlative approach uncovers previously inaccessible microscale relationships between transcript localization and collagen architecture, setting the foundation for a new paradigm in spatial biology. Beyond neuromuscular disease, the method is broadly applicable to a wide spectrum of tissues and pathologies, offering unique opportunities for discovery of spatially resolved biomarkers, insights concerning local fibrosis development and tissue regeneration, and more comprehensive microscale evaluation of the effects occurring in the tissue in response to therapeutic interventions involving transcript dynamics.

From a SHG imaging perspective, collagen microarchitecture can be described by computing a broader spectrum of quantitative parameters, compared to the sole fiber length, orientation and tortuosity. Indeed, signal intensity-based metrics, anisotropy or coherency indices, forward-to-backward (F/B) SHG ratios, and polarization-resolved measurements, provide complementary information on fibril packing, orientation dispersion, molecular order, and collagen maturation. To demonstrate the feasibility of our correlative multimodal framework, the minimal feature set we used (fiber length, orientation, and tortuosity) was sufficient to reveal spatial trends not present in bulk analyses, highlighting the added value of proximity-based interrogation enabled by the proposed correlative imaging workflow. The current approach is fully compatible with the integration of the cited additional metrics. Future studies assessing larger cohorts and biologically driven hypotheses will benefit from incorporating these additional SHG-related features, thereby enabling a more comprehensive and mechanistic characterization of collagen phenotypes in relation to transcript localization.

Therefore, this work proposes a versatile technological foundation to interrogate the interplay between gene activity and tissue structure. Its scalability, compatibility with advanced imaging and computational tools, and applicability across different diseases and biological structures make it a promising technology to advance both basic research and clinical translation.

## Methods

**Fig. 9:**
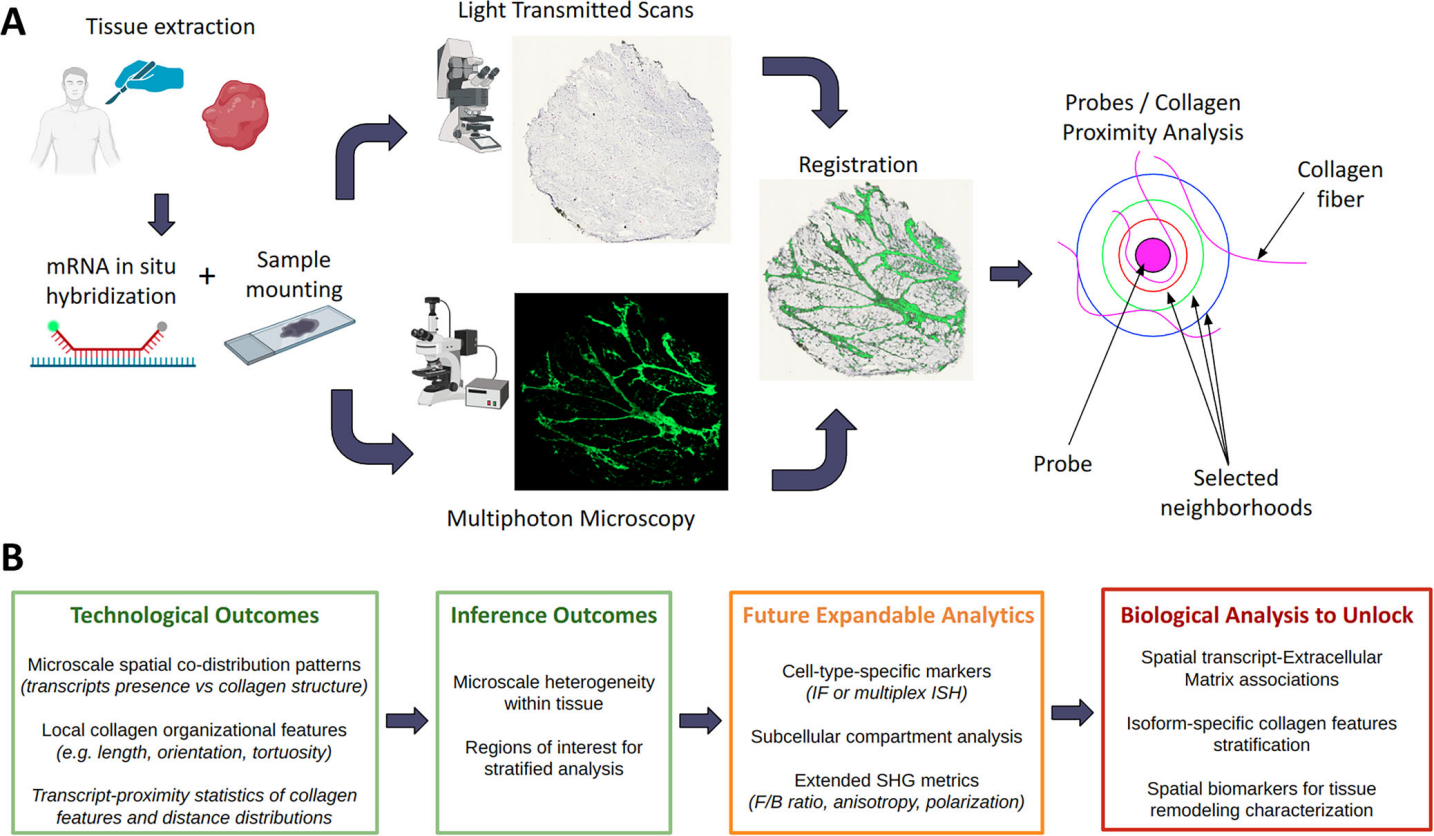
Schematic of the proposed workflow and conceptual framework. **A** RNAscope ISH assay was performed targeting exons 37–42 and exons 63–75 on human skeletal muscle biopsies. Each slide was imaged twice: first by brightfield microscopy to detect the RNAscope signals, and then by MPM to acquire label-free SHG signals from collagen. The resulting image pairs were subsequently registered to allow pixelwise correspondence. Finally, we performed a proximity-based spatial analysis to investigate whether local collagen architecture differs depending on the presence of transcript signal, enabling microscale assessment of molecular-structural correlations within the tissue. **B** In this conceptual framework, the green panels show the technical components currently implemented in this study (Technological Outcomes) and the corresponding inference derived from the analysis (Inference Outcomes). The orange panel highlights extensions that can be incorporated into the framework, while the red panel illustrates the new classes of biological investigation and insights that these future extensions could enable.

To provide a clear overview of our proposed methodology, we summarize the full imaging pipeline, from tissue extraction to image analysis, in Fig. 9A. Briefly, we collected tibial anterior muscle biopsies from 4 DMD patients carrying different mutations and one healthy control subject (wild-type: WT). On the mounted slides, we performed RNAscope ISH assay to localize *DMD* transcripts within the tissue using two different probes targeting exons 37–42 and exons 63–75, intercepting different dystrophin isoforms. The probe which targets exons 37–42 detects the full-length transcript Dp427m, while the other also recognizes the shorter isoform Dp71, leading to a partial overlap between the probe target regions. These dystrophin transcripts are purely muscle-expressed and were detected without concurrent cell-type-specific markers. Therefore, their cellular origin cannot be unambiguously resolved and the observed spatial proximity between RNAscope signals and collagen features should not be interpreted as direct evidence of transcript-ECM mechanistic coupling. We imaged each sample using both brightfield microscopy and MPM to obtain two full-section images. These images were spatially registered to ensure precise alignment. Brightfield images were analyzed to detect and localize transcript dots, while MPM images were processed to extract morphological features of individual collagen fibers.

Microscale spatial analysis was then performed using a proximity-based approach: for each transcript signal, concentric regions were defined around it to quantify collagen characteristics as a function of distance from the transcript site. This allowed us to assess whether local variations in collagen structure are associated with transcript presence. In the following sections, we provide a more detailed description of each step of the pipeline.

To provide a broader conceptual context for the proposed approach, we introduce a conceptual framework schematized in Fig. 9B. The green boxes show the modules implemented in this proof-of-concept, separating the pure technological components of the pipeline (Technological Outcomes) from the biological insights that can be drawn at the current development state (Inference Outcomes). The yellow module shows potential extensions of the proposed framework, including additional molecular readouts, imaging contrasts, or analytical strategies, which are not explored here but are technically compatible with the proposed approach. The red module highlights the broader classes of biological questions that such extensions could unlock, emphasizing the modular and extensible nature of the framework beyond the specific technical validation presented in this work.

### Enrolled subjects

Tibial anterior muscle biopsies were collected from four DMD patients and one healthy donor after informed consent for research purposes. All experimental protocols were approved by the University of Ferrara with ethical approval number 841/2020/Sper/AOUFe, in accordance with the Declaration of Helsinki. The enrolled individuals are listed in Table 1.

**Table 1:** List of enrolled subjects

Sample name	Phenotype	DMD mutation
WT	Healthy donor	/
DMD1	DMD	c.1264 G > T ex11 p.(Glu422*)
DMD2	DMD	Del ex 43
DMD3	DMD	Del ex 45
DMD4	DMD	Del ex 45–50

For each patient, the identified DMD mutations are reported.

### RNAscope assay

ISH was performed using RNAscope 2.5 HD Reagent assay Red (ACDbio). Polr2A (RNA Polymerase II Subunit A) was used as the positive control probe, while DapB (4-hydroxy-tetrahydrodipicolinate reductase) was used as the negative probe. Two independent inventoried probes targeting DMD transcripts were used:

- Probe Hs-DMD-Dp427m-E37-E42 contains 15 Z pairs and targets the nucleotides between 5400 and 6361 of NM_004006 (corresponding to exons 37–42 region).

- Probe Hs-DMD contains 20 Z pairs and targets the nucleotides between 9511 and 10,902 of NM_004006 (corresponding to exons 63–75 region). RNAscope assay was performed according to the manufacturer’s instructions with the following optimizations to enhance the specificity of the signal: (i) incubation of FFPE slides for 30 min at 60 °C, after the deparaffinization step; (ii) target retrieval step for 10 min; (iii) incubation of FFPE slides with Amp 5 for 60 min; (iv) pre-treatment protease digestion using Protease Plus.

### Multiphoton and brightfield microscopy

Images of the SHG signal from tissue sections of human skeletal muscle samples were acquired using an upright TriM Scope II multiphoton microscope (Miltenyi Biotech, Bielefeld, Germany). Excitation was provided by a tunable femtosecond laser (Cronus 2 P, Light Conversion, Vilnius, Lithuania) with a pulse duration of <160 fs, operating in the 680–960 nm range (first output channel, max power 1 W). The laser was tuned to 810 nm and operated at 10% output power, yielding an incident power of 13 mW at the sample plane for SHG excitation. Images were acquired using a Zeiss W Plan-Apochromat 20× water immersion objective (NA 1.0). Backscattered emission was directed through two longpass dichroic mirrors (cutoff wavelengths: 560 nm and 495 nm; Semrock) and detected by photomultiplier tubes (PMTs; H6780-01-LV, Hamamatsu, Herrsching am Ammersee, Germany) and GaAsP detectors (H7422-40-LV, Hamamatsu). Forward-scattered SHG signals were simultaneously collected by a separate PMT positioned below the stage, using a 1.4 NA condenser lens for focusing. Both forward and backward SHG emissions were filtered through a 405 ± 10 nm bandpass filter (BrightLine HD filters, Semrock; AHF Analysentechnik, Tübingen, Germany).

Image analysis was performed using the forward-scattered SHG. The entire tissue sections were imaged as two-dimensional mosaics. Each tile covered an area of 512 × 512 pixels, with a pixel dwell time of 1.63 µs and a resolution of 0.63 µm. Image acquisition included 2× line averaging and a 15% overlap between adjacent tiles. Mosaic stitching was performed using IMARIS software (Oxford Instruments).

Brightfield images for transcript signal detection were acquired using a Nikon Eclipse Ti2 microscope equipped with a 10× objective (NA = 0.3, working distance = 16 mm).

### Image registration

For each sample, the images of both brightfield and multiphoton microscopy were spatially aligned using a custom Python-based registration pipeline built on the SimpleITK library. This step ensured pixelwise correspondence between transcript detection (light transmitted scan) and collagen morphology (SHG signal). We reported the results in Section “Registration process”.

### Images independent processing and quantifications

Once the brightfield and multiphoton images were accurately registered, they were independently analyzed to extract specific information, although we show that their interpretive power is significantly enhanced when considered synergically. In the brightfield images, the key information corresponds to the localization of transcript-specific RNAscope signals, which appear as discrete reddish dots. Since this coloration resulting from the combination of red and blue channels is the only structure in the image with this chromatic profile, we implemented a simple thresholding algorithm to segment the transcript signals. Specifically, we computed a pixelwise ratio map using the RGB image channels, defined as G / (R + B), where a small epsilon value of 0.001 was added to the denominator to prevent division by zero. Pixels with a ratio below 0.4, indicating low green intensity, were classified as candidate probe dots. Subsequently, a binary mask was generated from this thresholded image, and connected circular components were identified as individual transcript signals. For each dot, we extracted morphological features including centroid position and area using region-based morphological operations. Detected RNAscope-positive dots were treated as spatial point features based on their centroid position. Dot area was used as a semi-quantitative descriptor of RNAscope signal extent and was not interpreted as a direct measure of absolute transcript abundance. No distinction was made between nuclear and cytoplasmic transcript localization, and subcellular compartmentalization was not considered in the present analysis. The statistical unit of analysis in this study is the individual biological sample (patient biopsy). To avoid pseudo-replication, collagen metrics reported in all the figures were averaged across all transcript-centered regions within each sample, and all reported proximity profiles represent sample-level aggregates. No dot-level statistical inference was performed across samples.

For the multiphoton images, we focused on quantifying the structural properties of collagen. Following the application of a γ correction of 0.75 and a 2 × 2 median filtering on the SHG signal, a pre-trained variational autoencoder^29^ was employed to segment and skeletonize the collagen fibers. Each identified fiber was assigned a unique ID, enabling us to track and analyze them individually. Based on the resulting segmentation, we generated derived maps in which each pixel of a skeletonized fiber was labeled with the corresponding value of a given morphological property.

Three primary structural features were computed for each fiber: (i) the length, computed by summing the pixel distances along the skeleton path, with diagonal steps weighted by the square root of 2 and axial steps by 1; (ii) the orientation, computed as the angle between the start and end points of the fiber’s major axis, constrained to the interval [0, π]; (iii) the tortuosity, defined as the ratio between the actual (curvilinear) length and the straight-line Euclidean distance between endpoints.

### Proximity analysis

To investigate whether the spatial proximity of transcript-associated probe signals correlates with local differences in collagen morphology, we developed a proximity-based analytical framework. For each sample, all segmented dots, identified from the brightfield image mask as described in Section “Images independent processing and quantifications”, were used as reference points. Around each dot centroid, we defined a series of concentric circular regions with increasing radii, ranging from 10 to 100 pixels (corresponding to approximately 6.3 to 63 µm). For each radius, we computed the mean values of the morphological properties of the collagen fibers (length, orientation, and tortuosity) lying in the region either partially or fully. These values were then averaged across all probe-associated regions within each sample. Mean values and standard errors of the mean (SEM) were plotted for each radius, allowing the identification of potential spatial trends and differences in collagen microarchitecture relative to transcript localization.

### Hardware and software

Processing and analysis were performed on a desktop workstation equipped with an AMD Ryzen 9 5900×12-core processor, 128 GB of RAM, and an NVIDIA GeForce RTX 3080 GPU with 12 GB of dedicated memory. Figure generation was performed using Fiji, an ImageJ package tailored for biological imaging. Plots and graphs were created using OriginPro 2025a (OriginLab Corporation). Custom Python scripts were developed and executed for image registration, segmentation, and feature extraction.

## Data Availability

Raw data are publicly available in a Zenodo repository^30^ at the link: https://zenodo.org/records/18220762.

## Code availability

Codes are publicly available in a Zenodo repository^30^ at the link: https://zenodo.org/records/18220762.
